# Dysregulation of hypoxia-inducible factor-1α (*Hif1α*) expression in the *Hmox1*-deficient placenta

**DOI:** 10.1016/j.placenta.2020.07.015

**Published:** 2020-09-15

**Authors:** Hui Zhao, Purnima Narasimhan, Flora Kalish, Ronald J. Wong, David K. Stevenson

**Affiliations:** a Department of Pediatrics, Stanford University School of Medicine, Stanford, CA, 94305, USA; b Department of Obstetrics and Gynecology, Stanford University School of Medicine, Stanford, CA, 94305, USA

**Keywords:** Angiogenesis, Spiral artery remodeling, Adverse pregnancy outcomes, Hypoxia, Antioxidant

## Abstract

**Introduction:**

Severe hypoxia exists in placentas during early pregnancy, with reoxygenation during mid-gestation. Hypoxia-inducible factor-1α (Hif1α), an oxygen sensor, initiates placental vascular development. We have shown that the placental vasculature in *Hmox1*-deficient (*Hmox1*^*+/−*^, Het) pregnancies is impaired, with morphological defects similar to *Hif1α*-deficient placentas.

**Materials and methods:**

Whole wild-type (WT) and Het mouse placentas were collected at E8.5 (1%–3% O_2_) and E9.5–15.5 (8%–10% O_2_). mRNA levels were determined using real-time RT-PCR or PCR arrays and protein levels using Western blot. Bone marrow-derived macrophages (BMDMs) from WT, Het, and *Hmox1* knockout (KO) mice, representing different Hmox1 cellular levels, were generated to study the role of *Hmox1* on *Hif1α* ′s response to hypoxia-reoxygenation and gestational age-specific placental lysates.

**Results:**

Hif1α was expressed in WT and Het placentas throughout gestation, with protein levels peaking at E8.5 and mRNA levels significantly upregulated from E9.5–E13.5, but significantly lower in Het placentas. Genes associated with angiogenesis (*Vegfa, Vegfr1, Mmp2, Cxcl12, Angpt1, Nos3*), antioxidants (*Sod1, Gpx1*), and transcription factors (*Ap2, Bach1, Nrf2*) were significantly different in Het placentas. In response to *in vitro* hypoxia-reoxygenation and to WT or Het placental lysates, *Hif1α* transcription was lower in Het and *Hmox1* KO BMDMs compared with WT BMDMs.

**Discussion:**

These findings suggest that deficiencies in *Hmox1* underlie the insufficient placental *Hif1α* response to hypoxia-reoxygenation during gestation and subsequently impair downstream placental vascular formation. Therefore, a dysregulation of *Hif1α* expression caused by any genetic defect or environmental influence in early pregnancy could be the root cause of pregnancy disorders.

## Highlights

• Expression of *Hif1α* in wild-type (WT) placentas is gestational age-dependent.
• *Hif1α* expression is reduced in *Hmox1*-deficient placentas.
• Expression of angiogenic genes is altered in *Hmox1*-deficient placentas.
• Hypoxia-reoxygenation induces a differential expression of *Hif1α* in cells.
• Adding placental lysates dysregulates expression of *Hif1α* in *Hmox1*-deficient cells.

## Abbreviations

ARNT: aryl hydrocarbon receptor nuclear translocator
BM: bone marrow
BMDMs: bone marrow-derived macrophages
CO: carbon monoxide
DFO: deferoxamine mesylate salt
Hmox: heme oxygenase
Het: *Hmox1^+/-^*
Hif: hypoxia-inducible factor
HRES: hypoxic response elements
KO: knockout
O_2_: oxygen
ROS: reactive oxygen species
SA: spiral artery
uNK: uterine natural killer
WT: wild-type

## 1 Introduction

In early pregnancy, a very critical window for placental development exists in both the human and mouse. During the 1st trimester, a severe hypoxic (1–3% oxygen [O_2_] or ~18 mmHg) microenvironment is present in the placenta due to the formation of a trophoblast plug within spiral arteries (SAs), which prevents the flow of maternal blood into the fetoplacental interface. During the 2^nd^ trimester, the plug dissolves and maternal blood can now enter the intervillous spaces, thereby increasing O_2_ levels to 8% (60 mmHg) [[1], [2], [3]]. This normal physiological phenomenon, while resembling an ischemia-reperfusion- (or hypoxia-reoxygenation)-like injury, is crucial for normal placental vascular development. A hypoxic environment is thought to be required for mediating the attachment of the blastocyst to the uterine wall in order to initiate placental proliferation of invasive trophoblast cells and maintain the stem cell state of trophoblasts by preventing their differentiation [[4], [5], [6]]. When O_2_ levels increase (8%), the trophoblast stem cells begin to differentiate into their invasive phenotype, which can migrate and invade into SAs, and stimulate maternal and fetal blood vessel growth [[7], [8], [9], [10]].

The primary cellular molecule that senses and responds to O_2_ tensions is hypoxia-inducible factor (Hif) [[11], [12], [13]]. Hif is a global regulator, capable of transcriptionally controlling the expression of more than 1000 genes by binding to DNA sequences that contain hypoxic response elements (HRES) [14]. Therefore, HIF can affect many cellular processes in response to hypoxia, such as angiogenesis, migration/invasion, erythropoiesis, and cell metabolism [15,16]. Hif is a heterodimer protein composed of two subunits: an alpha subunit with two isoforms (Hif1α and Hif2α) and a beta subunit [Hif1β or aryl hydrocarbon receptor nuclear translocator (Arnt)]. While Hif1β is insensitive to O_2_, Hif1α is degraded rapidly in the presence of O_2_.

Hif1α is found at high levels at 7–9 weeks of gestation when the O_2_ level in the placental microenvironment is 2%–3% and at low levels at 10–12 weeks of gestation when the O_2_ level is 8% to 10% [2,3]. Studies using *Hif1* knockout (KO) mice showed that the disruption of either the Hif1α or Hif1β subunit in fetal trophoblasts results in improper placental development, which is characterized by an insufficient chorion/allantois fusion and a diminished spongiotrophoblast and labyrinth layer [17,18]. Moreover, maternal Hif1α is also required for placentation by recruiting uterine natural killer (uNK) cells and trophoblasts into the maternal decidua and by affecting trophoblast function [17]. Therefore, insufficient *Hif1α* expression in either the maternal or fetal side of the placenta may be associated with impaired vascular development similar to that seen in preeclampsia [6].

Heme oxygenase (Hmox) is the enzyme that degrades heme to produce equimolar amounts of carbon monoxide (CO), iron, and biliverdin/bilirubin [19]. It is also a stress-response protein, since HO and its metabolites exhibit significant antioxidative [20], cytoprotective [21], pro-angiogenic [22], and anti-inflammatory [23] properties. Low expression of *Hmox1*, the inducible isoform, has been associated with a number of human pregnancy disorders [[24], [25], [26]]. In addition, our previous studies have shown that pregnant *Hmox1*-deficient (*Hmox1*^*+/−*^, Het) mice have preeclampsia-like features, such as hypertension and high sFlt 1 levels [27,28]. Het placentas also have insufficient SA remodeling and altered uNK cell differentiation and maturation [27,29,30]. Histological examination of Het placentas further revealed a markedly thinner spongiotrophoblast/giant cells layer compared with WT placentas [28,31]. Interestingly, many of these placental abnormalities resemble the phenotypes found in the pregnant *Hif1α*-deficient mouse.

Therefore, we hypothesized that a deficiency in *Hmox1* induces the dysregulation of *Hif1α*, which subsequently causes placental malformation and dysfunction. To this end, we compared Hif1α protein and mRNA levels throughout gestation between wild-type (WT) and Het placentas and investigated the response of Hif1α to either hypoxia-reoxygenation or placental lysates in bone marrow-derived macrophages (BMDMs) generated from WT, Het, and *Hmox1* KO mice.

## 2 Materials and methods

All studies were approved by Stanford University's Institutional Animal Care and Use Committee (Protocol #14525) and conducted in adherence to the National Institutes of Health Guidelines on the Use of Laboratory Animals.

### 2.1 Animals

Adult WT FVBn mice were purchased from Charles River Laboratories (Wilmington, MA). Adult female and male *Hmox1* Het pairs were used from our mouse colony. The original *Hmox1* KO (*Hmox1*^*−/−*^) mouse strain has a targeted deletion of a large portion of *Hmox1* gene, and was created on a C57BL/6 background. To establish our Het colony, C57BL/6 *Hmox1* mice were backcrossed with FVBn WT mice for >6 generations [28]. WT or Het mice were mated at 8–12 weeks of age. All animals were maintained and bred according to institutional guidelines of Stanford University. Gestational ages were calculated by the presence of a vaginal plug and recorded as E0.5. Het or *Hmox1* KO mice were genotyped using PCR as described previously [28].

### 2.2 Gene analyses

To create a customized PCR array, primers were chosen from the PrimerBank-MGH-PGA (Harvard University, Cambridge, MA) and validated by PCR screening. Specific genes were selected that encode for proteins: *Hmox1*, *Hmox2*, *Hif1α*, *Hif2α*, angiogenic factors (placental growth factor [*Pgf*]; stromal cell-derived factor 1 [*Cxcl12*]; vascular endothelial growth factor A [*Vegfa*]; matrix metalloproteinase-9 [*Mmp9*]; and matrix metalloproteinase-2 [*Mmp2*], angiopoietin-1 [*Angpt1*], nitric oxide synthase [*Nos2* and *Nos3*]), receptors (*Vegr1*, *Vegf 2*, C-X-C chemokine receptor 4 [*Cxcr4*]); antioxidant proteins (superoxide dismutase-1 [*Sod1*], −2 [*Sod2*]; glutathione peroxidase [*Gpx1*]), cyclin-dependent kinase inhibitor 1 (*Cdkn1A*, *P21*), transcription factors (*Sp1*, *Ap2*, *Nrf2*, and *Bach1*), and housekeeping genes (*Actb* and *Gapdh*) [32]. Primer sequences are shown in Table 1.

**Table 1:** Primer sequences.

	Primers
Hmox1	(F) 5′-AAGCCGAGAATGCTGAGTTCA-3′
	(R) 5′-GCCGTGTAGATATGGTACAAGGA-3′
Hmox2	(F) 5′-GGAGGGGGTAGATGAGTCAGA-3′
	(R) 5′-TCGGTCATGTGCTTCCTTGGT-3′
Hif1α	(F) 5′-TCTCGGCGAAGCAAAGAGTC-3′
	(R) 5′-AGCCATCTAGGGCTTTCAGATAA-3′
Hif2α	(F) 5′-GTGACATGATCTTTCTGTCGGAA-3′
	(R) 5′-CGCAAGGATGAGTGAAGTCAAA-3′
Pgf	(F) 5′-TGCTGGGAACAACTCAACAGAA-3′
	(R) 5′-TCTCCATGGGCCGACAGTAG-3′
Mmp2	(F) 5′-ACCTGAACACTTTCTATGGCTG-3′
	(R) 5′-CTTCCGCATGGTCTCGATG-3′
Mmp9	(F) 5′-GCAGAGGCATACTTGTACCG-3′
	(R) 5′-TGATGTTATGATGGTCCCACTTG-3′
Vegfa	(F) 5′-GCACATAGAGAGAATGAGCTTCC-3′
	(R) 5′-CTCCGCTCTGAACAAGGCT-3′
Vegfr1	(F) 5′-AGGGATAACAGGCAATTCTGC-3′
	(R) 5′-GTGCATCTCTATGAAAGGACTCC-3′
Vegfr2	(F) 5′-GTGATCCCAGATGACAGCCA-3′
	(R) 5′-GGTGAGCGCAGTGTGGTCC-3′
Cxcl12	(F) 5′-TGCATCAGTGACGGTAAACCA-3′
	(R) 5′-CACAGTTTGGAGTGTTGAGGAT-3′
Cxcr4	(F) 5′-GAAGTGGGGTCTGGAGACTAT-3′
	(R) 5′-TTGCCGACTATGCCAGTCAAG-3′
P21	(F) 5′-CGAGAACGGTGGAACTTTGAC-3′
	(R) 5′-CCAGGGCTCAGGTAGACCTT-3′
Angpt1	(F) 5′-ATCCCGACTTGAAATACAACTGC-3′
	(R) 5′-CTGGATGATGAATGTCTGACGAG-3′
Nos2	(F) 5′-GGAGCCTTTAGACCTCAACAGA-3′
	(R) 5′-TGAACGAGGAGGGTGGTG-3′
Nos3	(F) 5′-CCTTCCGCTACCAGCCAGA-3′
	(R) 5′-CAGAGATCTTCACTGCATTGGCTA-3′
Sod1	(F) 5′-AACCAGTTGTGTTGTCAGGA-C3′
	(R) 5′-CCACCATGTTTCTTAGAGTGAGG-3′
Sod2	(F) 5′-CCAAGGGAGATGTTACAACTCAG-3′
	(R) 5′-GGGCTCAGGTTTGTCCAGAA-3′
Gpx1	(F) 5′-AGTCCACCGTGTATGCCTTCT-3′
	(R) 5′-GAGACGCGACATTCTCAATGA-3′
Ap2	(F) 5′-TTTTTCAGCTATGGACCGTCAC-3′
	(R) 5′-GAAGTCGGCATTAGGGGTGTG-3′
Nrf2	(F) 5′-TCTTGGAGTAAGTCGAGAAGTGT-3′
	(R) 5′-GTTGAAACTGAGCGAAAAAGGC-3′
Sp1	(F) 5′-TGCAAACCAACAGATCATCCC-3′
	(R) 5′-TGACAGGTAGCAAGGTGATGT-3′
Bach1	(F) 5′-GCCTGAAGAGGTAACGGTTAAA-3′
	(R) 5′-GCACACTTCGTCAACATTGTC-3′
Actb	(F) 5′-AAGGAGATTACTGCTCTGGCTCCTA-3′
	(R) 5′-ACTCATCGTACTCCTGCTTGCTGAT-3′
Gapdh	(F) 5′-TGACCTCAACTACATGGYCTACA-3′
	(R) 5′-CTTCCCATTCTCGGCCTTG-3′

Total RNA was extracted from whole WT, Het, and *Hmox1* KO placentas (including deciduas, junction zones, and labyrinths) at different gestational ages using a Trizol Reagent (Thermo Scientific, Waltham, MA). cDNA was synthesized using a RT^2^ First Strand Kit (Qiagen, Redwood City, CA). Real-time RT-PCR was then performed using the RT^2^ Real-Time SYBR Green/ROX PCR Master Mix (Qiagen) on a Stratagene Mx3005P QPCR system (Agilent Technologies, Palo Alto, CA). Data were then analyzed using the ΔΔCt method, normalized to *Actb* and *Gapdh*. Fold change in gene expression over control levels were then calculated.

### 2.3 Western blots

Whole placental samples were sonicated and prepared as previously described [33]. 2 mM deferoxamine mesylate salt (DFO, Sigma-Aldrich, St. Louis, MO) was then added to prevent Hif1α degradation [34]. 100 μg of sonicate was boiled for 10 min in protein loading buffer and separated using 12% SDS-PAGE gel electrophoresis (BioRad, Hercules, CA). Proteins were transferred to a PDVF membrane (Bio-Rad) using a semidry transblotter (Bio-Rad). The membrane was probed with polyclonal antibodies raised against Hif1α (Invitrogen, Carlsbad, CA) and Actb (Santa Cruz Biotechnology, Santa Cruz, CA). Signals were detected using a SuperSignal™ West Pico PLUS chemiluminescent substrate (Thermofisher, Waltham, MA) and band intensities were quantitated using Image Studio Lite software (LI-COR Biosciences, Lincoln, NE). Coomassie blue staining was performed to confirm equal loading of samples.

### 2.4 *In vitro* establishment and stimulation of BMDMs

BMDMs were generated from bone marrow (BM) cells isolated from WT, Het, and *Hmox1* KO mice as described [32]. In brief, 1 × 10^7^ BM cells in 10 ml media (10^6^ cells/ml) were seeded onto 100-mm Petri-dish plates (Fisher Scientific, Pittsburgh, PA) and maintained in 20% L929 conditional RPMI medium with β-mercaptoethanol. At day 3, half of the media was replaced with fresh media. At day 7, attached cells were treated with IL-4 (20 ng/ml, Miltenyi Biotec, Sunnyvale, CA) to differentiate M1 cells into M2a cells.

### 2.5 Study design

#### 2.5.1 Hypoxia studies

M2a cells were exposed for 24 h at an O_2_ concentration of 21%, 5%, 1%, or 1%–21% using a hypoxia incubator (Thermo Scientific).

#### 2.5.2 Placental lysate studies

Placentas from pregnant WT and Het mice at E8.5 or E10.5 were collected in RPMI medium. Tissues were homogenized, centrifuged, and filtered through 0.22-μm columns to yield crude lysates for treatment of BMDMs.

### 2.6 Statistical analyses

For comparisons of experimental groups, one-way ANOVAs were first performed for each set of experiments to determine significant differences when P < 0.05. To determine differences between individual experimental and control groups, Dunnett's tests were used which adjusts for multiple comparisons using the same control group.

## 3 Results

### 3.1 Expression of *Hif1α* in WT placentas is gestational age-dependent

**Fig. 1:**
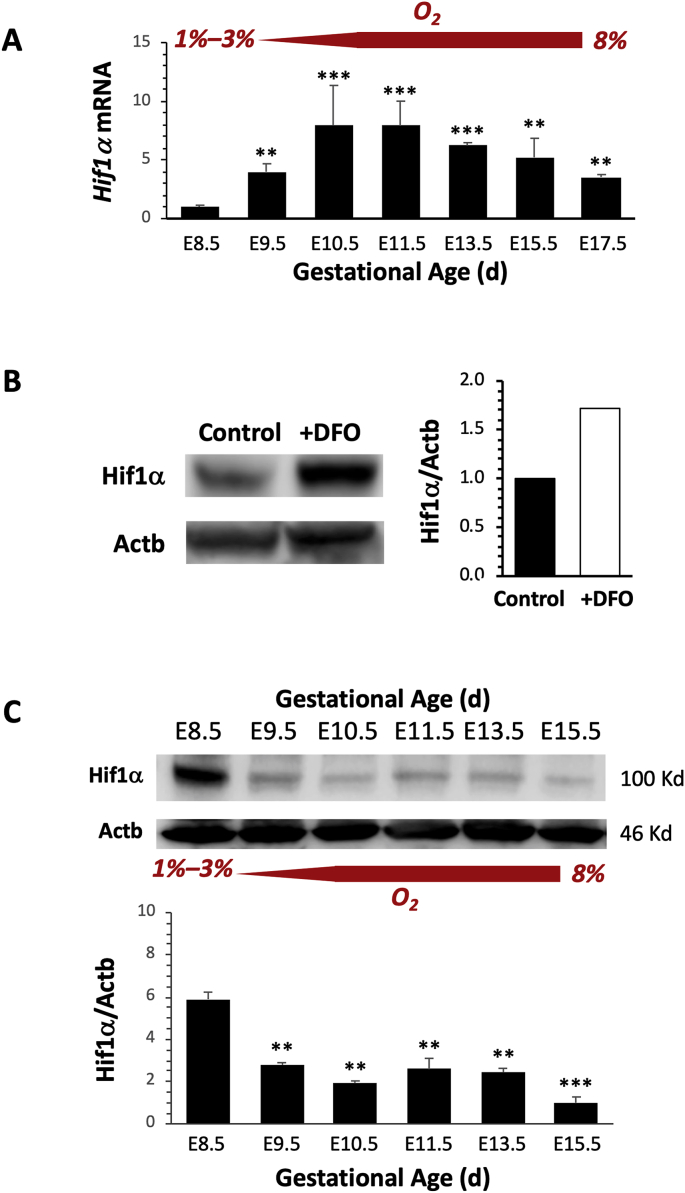
**Expression of *Hif1α* is gestational age-dependent.** (**A**) *Hif1α* mRNA levels at various gestational ages (n = 3 at each age) were measured using real-time RT-PCR and normalized to *Gapdh* and *Actb* levels. Significant upregulation was observed when the placental oxygen (O_2_) microenvironment increased from 3% to 8% at E9.5 to 10.5. (**B**) Addition of DFO to placental samples (white bar) helped to preserve Hif1α protein from degradation under ambient oxygen conditions as compared with control (no DFO) samples (black bar) as shown by Western blots. (**C**) Representative images of Western blots of Hif1α and Actb protein levels (top panel). Band intensities were quantified and normalized to Actb (n = 3). *P < 0.05; **P < 0.01; ***P < 0.005.

To investigate whether *Hif1α* expression in WT placentas change throughout gestation, we collected placentas at E8.5 to E17.5 and measured *Hif1α* mRNA levels using real-time RT-PCR. Significant upregulation of *Hif1α* mRNA was observed beginning at E9.5, when O_2_ levels are at 8% compared with O_2_ levels of 1%–3% at E8.5. Levels peaked between E10.5 to E11.5, and gradually decreased thereafter (Fig. 1A).

To quantitate Hif1α protein levels, WT placentas were collected at E8.5 to E15.5 and processed for Western blots. To prevent Hif1α degradation during ambient O_2_ exposure, DFO, an iron chelator, was added to the samples during homogenization [35]. For samples without DFO (controls), lysates were prepared as fast as possible to limit exposure to air (normally within 1 min or less) before the addition of loading buffer. We found that addition of DFO yielded up to 70%–80% more Hif1α protein compared with control samples (Fig. 1B). Therefore, for all placental samples processed for Western blot analysis, DFO was added. We found that Hif1α protein levels in WT placentas peaked at E8.5 when the placenta was under severe hypoxia, and decreased from E9.5 to E15.5 when O_2_ levels increased (Fig. 1C).

### 3.2 *Hif1α* expression is reduced in *Hmox1*-deficient placentas

**Fig. 2:**
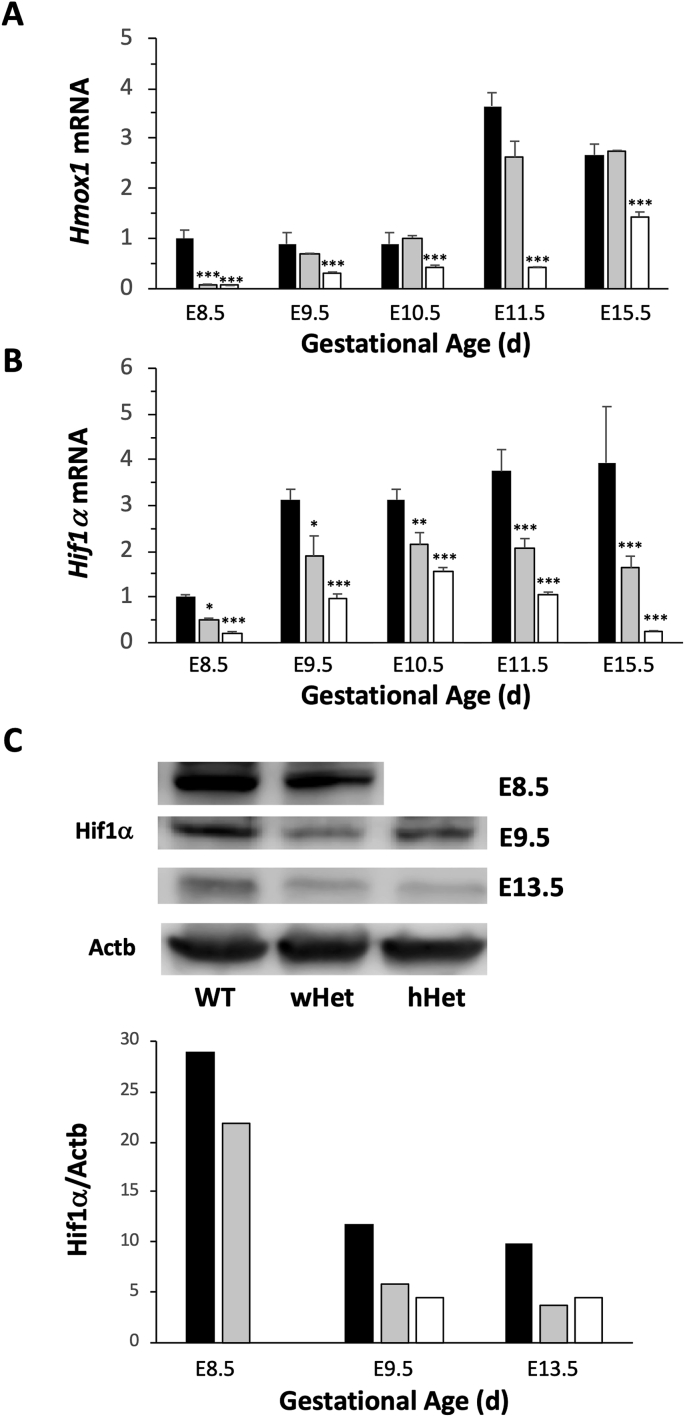
**Het placentas have significantly reduced *Hif1α* expression.** (**A**) *Hmox1* deficiency in placentas from Het pregnancies with WT fetuses (wHet, gray bars, n = 3 at each age) or Het fetuses (hHet, white bars, n = 3 at each age) was confirmed by measuring mRNA levels using real-time RT-PCR, normalized to *Gapdh* and *Actb* levels, and compared with those fetuses from WT pregnancies (WT, black bars, n = 3 at each age). (**B**) *Hif1α* mRNA expression was measured in placentas at various gestational ages harvested from WT pregnancies (WT, black bars, n = 3 at each age), Het pregnancies with WT fetuses (wHet, gray bars, n = 3 at each age) or Het fetuses (hHet, white bars, n = 3 at each age). *Hif1α* expression was normalized to *Gapdh* and *Actb* levels. (C) Hif1α protein levels in placentas at various gestational ages harvested from Het pregnancies with WT fetuses (wHet, gray bars) or Het fetuses (hHet, white bars) compared with placentas from WT pregnancies (black bars) are shown by Western blots. Hif1α expression was normalized to Actb levels. Due to the extremely small size of fetuses at E8.5, standard genotyping could not be performed. Therefore, *Hmox1* mRNA levels were used to differentiate wHet and hHet placentas for mRNA studies, but was not feasible for Western blots. *P < 0.05; **P < 0.01; ***P < 0.005.

In Het pregnancies where Het females are mated with Het males, either WT or Het, but no *Hmox1* KO, fetuses are present as observed previously [27,28]. After genotyping fetuses from Het pregnancies, placentas were then designated as follows: wHet for placentas from WT fetuses; hHet for placentas from Het fetuses; and WT for placentas from WT to WT breedings. *Hmox1* deficiency was confirmed using RT-PCR, which showed significant reduction of *Hmox1* in both wHet and hHet placentas at E8.5 compared with age-matched WT placentas (Fig. 2A). At E85, placentas are primarily composed of maternal uteri tissue or decidua. However, *Hmox1* was significantly reduced only in hHet, but not in wHet, placentas at E10.5 to E15.5, when placentas are primarily composed of labyrinth tissue, the majority of which originate from the fetus, in wHet placentas.

When we compared *Hif1α* mRNA expression in WT and Het placentas during pregnancy, we found a similar gestational age-dependent profile with an upregulation of transcription beginning at E9.5 and peaking around E10.5 to E11.5 (Fig. 2B). However, *Hif1α* mRNA levels in wHet placentas were significantly lower than those in WT placentas. Furthermore, *Hif1α* mRNA levels in hHet placentas were even lower than those in wHet placentas, which suggests that a reduction *Hif1α* mRNA is associated with *Hmox1* deficiency. These findings were confirmed by quantification of Hif1α protein levels using Western blots, where we found decreased Hif1α protein levels in wHet and hHet placentas compared with those in WT placentas at E8.5, E9.5, and E13.5 (Fig. 2C).

### 3.3 Expression of angiogenic genes is altered in *Hmox1*-deficient placentas

**Fig. 3:**
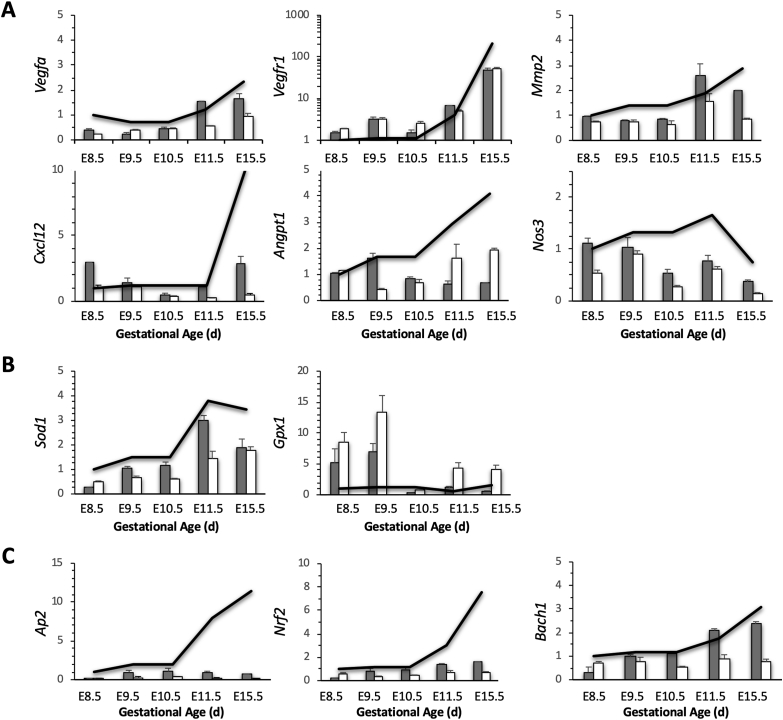
**Differential gene expression profiles**. mRNA levels of (**A**) angiogenic-associated genes; (**B**) antioxidant genes; and (**C**) transcription factors were measured using PCR arrays and compared between WT (black line, n = 3), wHet (gray bar, n = 3), and hHet (white bar, n = 3) placentas. All levels were normalized to *Gapdh* and *Actb* levels.

Using a custom-made PCR-array [32], we compared genes that are related to hypoxia-reoxygenation between WT and Het placentas. Expression of several genes associated with angiogenesis in wHet or hHet placentas were either suppressed (such as *Vegfa*, *Mmp2*, *Cxcl12*, *Angpt1*, and *Nos3*) or induced (*e.g., Vegfr1*) compared with WT placentas. The alterations in *Vegfa*, *Vegfr1,* and *Mmp2* occurred early in pregnancy (E8.5 to E10.5), while changes in *Cxcl12*, *Angpt1*, and *Nos3* were primarily observed at mid-to-late gestation (E11.5 to E15.5) (Fig. 3A). In addition, two antioxidant genes, *Sod1* and *Gpx1*, were decreased or increased, respectively, especially in hHet placentas (Fig. 3B). Expression of transcription factors, *Ap2* and *Nrf2*, were reduced in both wHet and hHet placentas, while *Bach1* was down-regulated only in hHet, but not in wHet placentas (Fig. 3C). *Hmox1* deficiency had no significant effects on the expression of *Hif2α* or *Pgf* (data not shown).

### 3.4 Hypoxia-reoxygenation induces the differential expression of *Hif1α* in BMDMs

**Fig. 4:**
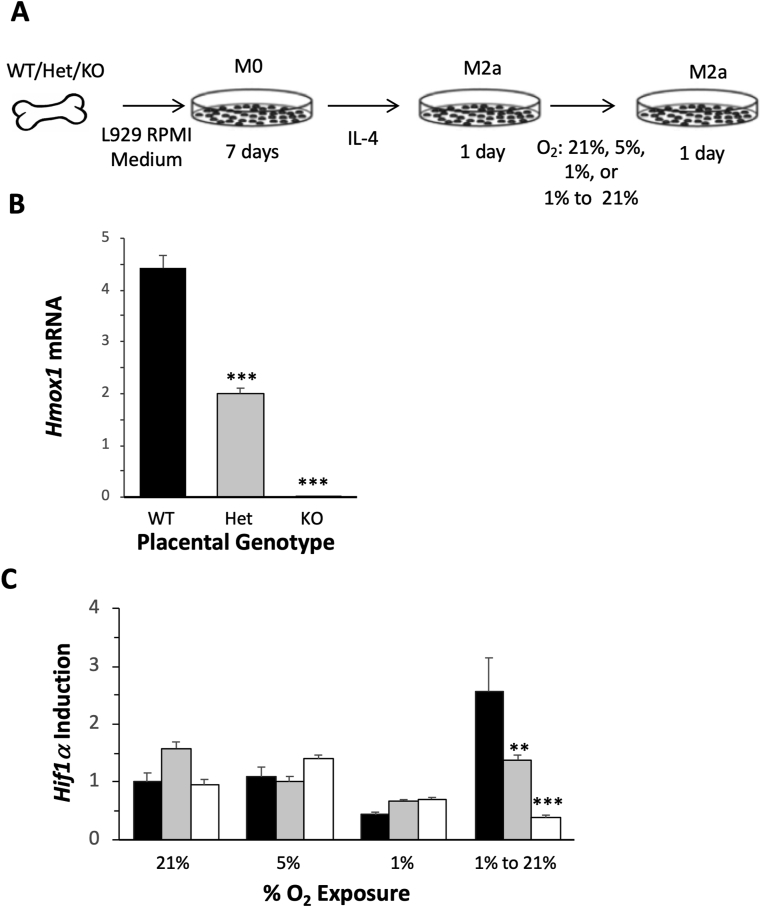
**Differential expression of *Hif1α* in WT, Het, or *Hmox1* KO cells in response to hypoxia-reoxygenation exposures.** (**A**) Graphic of our study design. (**B**) *Hmox1* expression in WT (black bar, n = 3), Het (gray bar, n = 3), and *Hmox1* KO (white bar, n = 3) M2a cells. (**C**) *Hif1α* mRNA levels were measured after WT (black bars, n = 3 at each O_2_ level), wHet (gray bars, n = 3 at each O_2_ level), and hHet (white bars, n = 3 at each O_2_ level) M2a cells were exposed to 21%, 5%, 1%, or 1 to > 20% O_2_ levels. *Hmox1* and *Hif1α* expression levels were normalized to *Gapdh* and *Actb* levels. *P < 0.05; **P < 0.01; ***P < 0.005.

To further investigate whether *Hmox1* deficiency underlies the differential expression of *Hif1*α in response to hypoxia-reoxygenation, we first generated BMDMs from non-pregnant WT, Het, and *Hmox1* KO mice and then treated these cells with IL-4 to differentiate these M0 cells into M2a cells. These cells were further exposed to different oxygen tensions: 21%, 5%, 1%, and 21% after 1% pretreatment. Our experimental design is outlined in Fig. 4A. After 24 h, cells were harvested and mRNA was isolated. *Hmox1* deficiency in Het and *Hmox1* KO cells were confirmed by RT-PCR (Fig. 4B). When cells were exposed to 21%, 5%, or 1% O_2_, no significant differences in *Hif1α* mRNA levels were observed between WT, Het, or *Hmox1* KO cells. However, when cells that were initially exposed to O_2_ at 1% and then re-exposed to 21% to mimic the hypoxia-reoxygenation environment observed in early pregnancy, induction of *Hif1α* transcription was found to vary significantly between genotypes (Fig. 4C). Compared with mRNA levels at 1% O_2_, *Hif1α* was significantly upregulated in WT cells (~6-fold), but less increased in Het cells (~2-fold). However, *Hif1α* was not induced in *Hmox1* KO cells (~0.5 fold), suggesting that the response of *Hif1α* to hypoxia-reoxygenation is regulated by the level of *Hmox1* expression.

### 3.5 Addition of placental lysates dysregulates expression of *Hif1α* in BMDMs

**Fig. 5:**
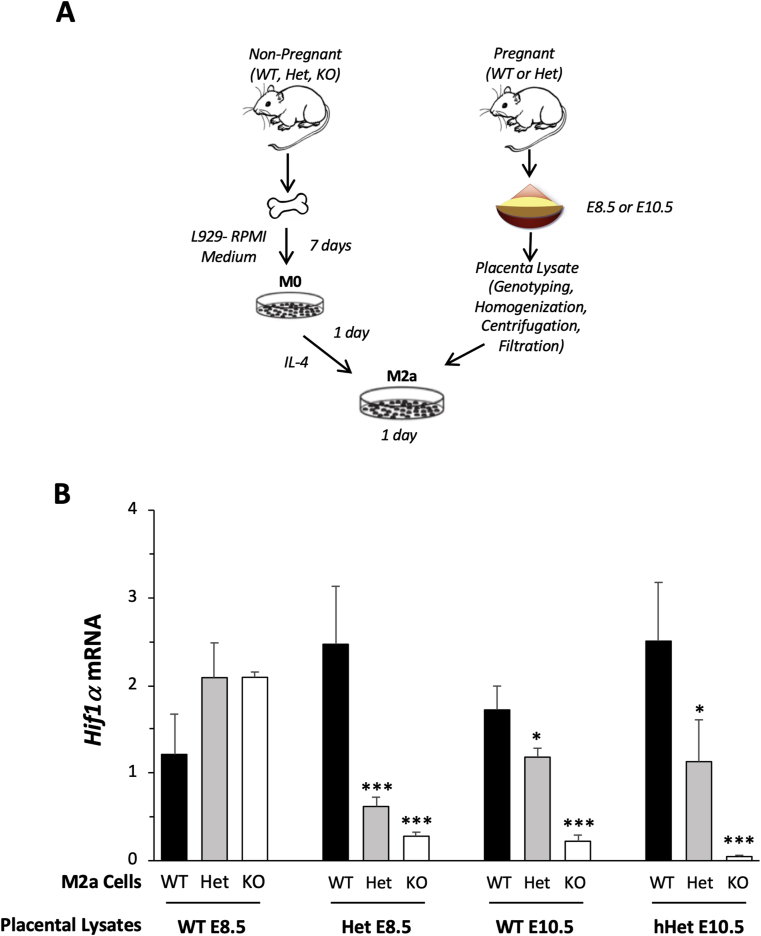
**Differential *Hif1α* expression in response to the addition of placental lysates.** (**A**) Graphic of our study design. (**B**) M2a cells with different *Hmox1* genetic backgrounds (WT, black bars, n = 3; wHet, gray bars, n = 3; and hHet, white bars, n = 3) were treated with WT or Het lysates prepared from placentas harvested at E8.5 and E10.5. *Hif1α* mRNA levels were measured using real-time RT-PCR after 24 h of treatment and normalized to *Gapdh* and *Actb* levels. *P < 0.05; **P < 0.01; ***P < 0.005.

To investigate whether the dysregulation of *Hif1α* expression in Het placentas was due to insufficient *Hmox1* expression in the cells or due to the *Hmox1* deficiency-induced alterations in the placental microenvironment, we generated BMDMs from WT, Het, or *Hmox1* KO mice and exposed these cells with lysates prepared from WT or Het placentas harvested at two gestational ages, E8.5 and E10.5, when O_2_ levels are low and high, respectively (Fig. 5A).

*Hif1α* induction was expressed as the relative fold change in *Hif1α* levels from the placental-lysate-treated cells over basal levels from non-treated control M2a cells. Differential expression of *Hif1α* in WT, Het, or *Hmox1* KO M2a cells was not found after treatment with E8.5 WT placental lysates, but observed after treatment with WT placental lysates at E10.5 or Het placental lysates from E8.5 or E10.5, in a *Hmox1* dose-dependent fashion (Fig. 5B). These data suggested that both cellular *Hmox1* levels and extracellular factors could contribute to *Hif1α* expression in the placenta.

## 4 Discussion

We found that the severe hypoxic environment in the placenta during early mouse pregnancies can trigger *Hif1α* signaling, similar to that reported for human pregnancies [36]. *Hif1α* is regulated both at the transcriptional as well as post-translational levels. The half-life of Hif1α protein is short, being only about 5 min in ambient air [37], and likely contributes to the lack of correlation between *Hif1α* mRNA and Hif1α protein levels [36], as we have found in our studies (Fig. 1). We found that the severe hypoxic placental microenvironment at E8.5 sustained a high Hif1α protein level due to its low degradation at this O_2_ level. In contrast, higher O_2_ levels during E9.5 to 10.5 decreased protein levels, but increased *Hif1α* transcription (Fig. 1). This *Hif1α* transcriptional profile in the mouse placenta is consistent with the findings reported in human placental studies by Ietta et al., where Hif1α protein levels are highest around 7 to 10 gestational weeks when severe hypoxia is present, while mRNA levels peak between 14 to 18 gestational weeks when reoxygenation occurs [36].

The upregulation of *Hif1α* mRNA at E9.5 to E11.5 apparently was likely not due to hypoxia, but possibly due to the presence of other factors, such as reactive oxygen species (ROS), growth factors, and inflammatory cytokines. Due to their ability to induce *Hif1α* expression, these factors are also called “pseudo-hypoxia factors” [15]. We speculate that at the end of the 1^st^ trimester, placental O_2_ levels rapidly increase, which can then lead to a transient period of oxidative stress as well as increased inflammatory cytokine levels. This is supported by our observations that certain antioxidant genes, such as *Sod1* and *Hmox1*, significantly increase from E9.5 to E11.5 (Fig. 3B). In addition, when we treated M2a cells with different fractions of placental lysates, which were separated based on molecular weight, the highest induction of *Hif1α* was found in the < 3-kd fraction (unpublished data), which contains the highest levels of ROS.

Even though *HIF1α* has been shown to regulate *Hmox1* expression [38], the effect of *Hmox1* levels on *Hif1α* expression has not as yet been elucidated. Here, we show evidence that *Hmox1* deficiency could induce the dysregulation of *Hif1α* in the placenta. Compared with WT placentas, significantly lower *Hif1α* mRNA and Hmox1 protein levels were found in Het placentas throughout all gestational ages (Fig. 2). This finding may possibly explain why Het placentas, especially hHet placentas, share similar morphological defects with those placentas from *Hif1α*-deficient pregnancies [39]. Since Hif1α also triggers the differentiation of trophoblast stem cells to an invasive phenotype [4], it is not surprising that *Hmox1-*deficient placentas have insufficient SA remodeling and impaired vascular development in the labyrinth due to lower *Hif1α* levels [27].

Hif1α is a global regulator that controls the expression of many genes, including *Vegfa* and *Vegfr1* [32], which are critical for placental vasculogenesis and branching angiogenesis in early pregnancy. In our study, we found that placental *Vegfa* and *Vegfr1* were significantly altered as early as E8.5 in *Hmox1-*deficient placentas (both wHet and hHet) compared with WT placentas (Fig. 3A), suggesting they may be controlled directly by *Hif1α*. Interestingly, no significantly changes were found in *Hif2α* as well as *Pgf*, another very important placental angiogenic factor that binds to *Vegfr*. These results support the finding that *Pgf* in trophoblasts is mostly regulated by *Hif2α* [40]. In addition, we also observed that the levels of other angiogenic-associated genes, such as *Mmp2*, *Cxcl12*, *Angpt1*, and *Nos3*, were decreased, but at a relatively later stage (E10 or so) (Fig. 3A), suggesting an indirect control by *Hif1α*, via a secondary event due to either a deficiency in *Vegf* expression or an impairment of SA remodeling.

To further elucidate whether the decrease in *Hif1α* expression in *Hmox1* deficiency placenta is primarily due to the manipulation of the cellular molecular signaling or the changes of placental microenvironment, we used cultured WT, Het, and *Hmox1* KO BMDMs treated with different O_2_ tensions or lysates from WT or Het placentas. BM and its derived cells (BMDMs) are relatively easier to collect, differentiate, and maintain compared with other primary cells, such as trophoblasts or endothelial cells. In these studies, we found that a deficiency of *Hmox1* (either Het or KO) alone is not associated with the differential *Hif1α* expression at either normoxia or hypoxia; but only at the transition state from hypoxia to reoxygenation (Fig. 4C). It also explains the results of our placental lysate studies, which showed that lysates at E10.5, when the placentas are re-exposed to increased O_2_ levels caused a dysregulation of *Hif1α* in *Hmox1-*deficient M2a cells (Fig. 5B). In addition, Het placental lysates, but not WT, at E8.5 also induced the dysregulation in Het or *Hmox1* KO BMDM cells, suggesting abnormal tissue stress in the Het placenta in early pregnancy. Taken together, these data suggested that failure to respond to the oxidative stress in *Hmox1*-deficient cells is associated with *Hif1α* suppression. Even though the molecular mechanism that cross-links *Hmox1* and *Hif1α* is not yet understood, we speculate that it might be associated with oxidative stress and mitochondria function [[41], [42], [43], [44], [45], [46]].

We are also aware of the limitations of this study. Only whole placental tissues were used for measurements of *Hif1α* expression, and therefore the identification of the specific cell type(s) affected by *Hmox1* deficiency was not possible. Previous work has shown that *Hmox1* is expressed primarily by trophoblast cells, such as invasive trophoblasts and spongiotrophoblast cells [47]. Thus, we speculate that a down-regulation of *Hif1α* is caused by a deficiency in *Hmox1*, which could also occur in these trophoblast cells, and are well known for their role in SA remodeling, the failure of which is likely a contributing cause of preeclampsia.

The establishment of the fetomaternal interface is intricately regulated by O_2_ tensions in early pregnancy, followed by an increased in vascular formation via angiogenesis in later pregnancy. Therefore, the dysregulation of *Hif1α*, resulting in either a down- or upregulation, would have detrimental effects on SA remodeling and subsequent abnormal fetoplacental vascular formation [48]. Our findings reveal that a deficiency in *Hmox1* could alter *Hif1α* expression in response to O_2_ tensions in the placental microenvironment and cause an alteration of the downstream genes associated with angiogenesis. Our model has provided an example of how pregnancy disorders can be affected by a dysregulation of *Hif1α* induced by other genetic mutations. Further research is needed to identify other gene or combinations of genes that can also affect *Hif1α* expression in response to hypoxia-reoxygenation, improving our understanding of placental pathology and possibly guiding therapeutic approaches to various pregnancy disorders involving placental vascular maldevelopment.

## Statement of financial support

This work was supported by the Prematurity Research Fund; the 10.13039/100000912March of Dimes Prematurity Research Center at Stanford University; the Charles B. and Ann L. Johnson Research Fund; the Christopher Hess Research Fund; the Providence Foundation Research Fund; the Roberts Foundation Research Fund; the Stanford Maternal and Child Health Research Institute; and the 10.13039/100000865Bill and Melinda Gates Foundation.

## Declaration of competing interest

All the authors confirm that they have the guidance on competing interests and none of the authors have any competing interests in the manuscript.
